# Supplementary material for: Causal Relationship between Aging and Anorexia Nervosa: A White-Matter-Microstructure-Mediated Mendelian Randomization Analysis
Source: Biomedicines. 2024 Aug 16;12(8):1874. doi: 10.3390/biomedicines12081874 (PMC11351342; doi:10.3390/biomedicines12081874)
Supplement: Supplementary file 1 [file biomedicines-12-01874-s001.zip › Supplementary Figure/Figure S8/Leaveoneout plot of MR analysis for TL to ubm-b-1786.pdf]

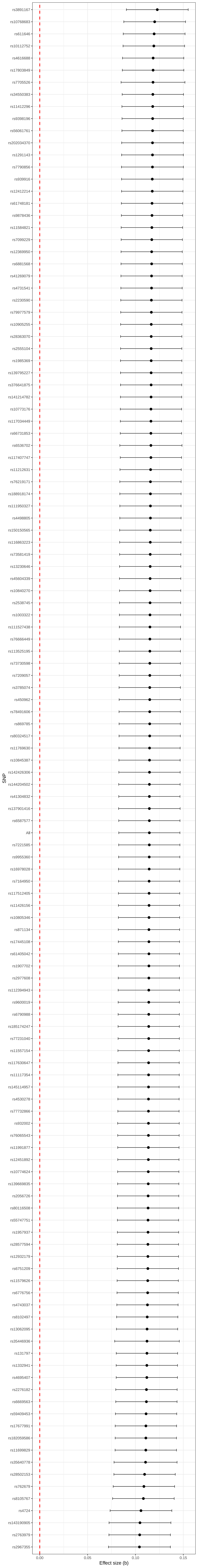

rs3891167
rs10768683
rs611646
rs10112752
rs4616688
rs17803849
rs7705526
rs34550383
rs11412296
rs9398196
rs56061761
rs202034370
rs1291143
rs7790856
rs939916
rs12412214
rs61748181
rs9878436
rs11584821
rs7099229
rs12369950
rs6881568
rs41269079
rs4731541
rs2230590
rs79977579
rs10905255
rs28363070
rs2555104
rs1985369
rs139795227
rs376641875
rs141214782
rs10773176
rs117034449
rs66731853
rs6536702
rs117407747
rs11212631
rs76219171
rs188918174
rs111950327
rs4498805
rs150150565
rs116863223
rs73581419
rs13230646
rs45604339
rs10840270
rs2538745
rs1003322
rs111527438
rs76666449
rs113525185
rs73730598
rs7209057
rs3785074
rs450962
rs78491606
rs869785
rs80324517
rs11769630
rs10845387
rs142426306
rs144204502
rs41304832
rs137901416
rs6587577
All
rs7221585
rs9955360
rs16978028
rs7164950
rs117512405
rs11426156
rs10805346
rs871134
rs17445108
rs61405042
rs1907702
rs2977608
rs112394943
rs9600019
rs6790988
rs185174247
rs77231040
rs11557154
rs117630647
rs11117354
rs145114957
rs4530278
rs77732866
rs932002
rs76065543
rs11991877
rs12451892
rs10774624
rs139669835
rs2056726
rs80116508
rs55747751
rs1957937
rs28577594
rs12932179
rs6751209
rs11579626
rs6776756
rs4743037
rs8102497
rs13062095
rs35446936
rs131797
rs1332941
rs4695407
rs2276182
rs6669563
rs59409453
rs17677991
rs182059586
rs11699829
rs35640778
rs28502153
rs762679
rs8105767
rs4724
rs143190905
rs2763979
rs2967355
SNP
0.00
0.05
0.10
0.15
Effect size (b)
